# Evaluation of fluorescence excitation transfer immunoassay for the measurement of plasma cortisol

**DOI:** 10.1155/S1463924686000172

**Published:** 1986

**Authors:** J. Calvin, K. Burling, R. S. Campbell, S. A. P. Chubb, C. P. Price

**Affiliations:** Department of Clinical Biochemistry Addenbrooke's Hospital Cambridge CB2 2QR UK


Journal of Automatic Chemistry/*Journal of Clinical Laboratory Automation*, Vol. 8, No. 2 (April–June 1986), pp. 80–84

### Evaluation of fluorescence excitation transfer immunoassay for the measurement of plasma cortisol

**J. Calvin, K. Burling, R. S. Campbell, S. A. P. Chubb and C. P. Price**

*Department of Clinical Biochemistry, Addenbrooke's Hospital, Cambridge CB2 2QR, UK*

Fluorescence excitation transfer immunoassay is a form of homogeneous assay suitable for the measurement of both large and small molecules.

Förster in 1948 [1] demonstrated that energy can be transferred through space from a fluorescent donor to an acceptor molecule; immunoassays based on this observation have now been developed. In fluorescence excitation transfer immunoassay, antibody is labelled with acceptor molecule or 'quencher', and the antigen with fluorescer molecules. When the antigen and antibody bind, the fluorescer and quencher molecules are brought into close proximity and the fluorescence decreases. Antigen in the sample competes with the labelled antigen for antibody sites. Thus a high analyte concentration results in a low level of quenching and vice versa [2].

The extent of quenching partly depends on the distance between the quencher and fluorescer [3]; the quenching rapidly decreasing with increasing distance. For this reason antibody rather than antigen is labelled with quencher. If the antibody was labelled with fluorescer, molecules bound at sites distant from the antigen-antibody binding site may not be effectively quenched by labelled antigen. The quencher must absorb at the wavelength of the fluorescence emission; the higher the molar absorptivity the greater the quenching efficiency. For a homogeneous immunoassay the number of quencher molecules coupled to the antibody is limited by the effect on the protein solubility and by any background fluorescence due to the quencher itself [2].

The fluorescence excitation transfer immunoassay for the measurement of plasma cortisol uses a fluorescein derivative (4,5-dimethoxyfluorescein) as the quencher molecule. This compound does not contribute to the background fluorescence. The hapten, cortisol, is labelled with 2,7-dimethoxy-4,5-dichlorofluorescein, a dye which absorbs at 540 nm and emits at 560 nm [4].

This paper reports on the performance of the fluorescence excitation transfer immunoassay technique for the measurement of plasma cortisol.

### Materials and methods

*Instrumentation*

The Syva Advance was used to assay samples by fluorescence excitation transfer immunoassay. The fluorimeter was calibrated on each working day, according to the manufacturer's recommendations.

*Reagents*

Cortisol kits were obtained from Syva UK (Syntex House, Maidenhead, Berkshire, UK).

*Plasma pools*

Plasma pools containing three levels of analyte were prepared from patients' samples. The levels were chosen for clinical relevance. The pools were aliquoted and stored at −20 °C.

### Experimental procedures and results

*Imprecision*

Samples of each of the plasma pools were analysed on 20 consecutive working days to determine the between-batch precision. The data are shown in table 1.

A sequence of low (L), medium (M) and high (H) level samples were run on three separate occasions during the period of 20 days, permitting the calculation of within-batch precision on 10 results. The imprecision ranged from 3·4 to 5·8% (low), 1·8 to 3·2% (medium) and 1·7 to 3·1% (high).

On one occasion two sequences were run on consecutive carousels to facilitate calculation of the within-batch imprecision using 20 readings. The data are shown in table 1.

*Table 1. Imprecision.*

| | Cortisol (nmol/l) | | |
|---|---|---|---|
| Within batch | | | |
| Mean | 267 | 528 | 891 |
| S.D. | 12·3 | 20·1 | 28·5 |
| C.V. (%) | 4·6 | 3·8 | 3·2 |
| *N* | 20 | 20 | 20 |
| Between batch | | | |
| Mean | 204 | 431 | 773 |
| S.D. | 13·8 | 22 6 | 30·2 |
| C.V. (%) | 6·8 | 5·2 | 3·9 |
| *N* | 20 | 20 | 20 |

*Carry-over*

Using the results obtained from the within-batch imprecision studies carry-over was calculated according to the Dixon formula [5].

The maximum carry-over was 2·6%.

*Recovery of added analyte*

Cortisol (Sigma Chemical Company, Poole, Dorset, UK) was dissolved in ethanol to give a stock solution of 11 μmol/l. Five patient samples were spiked with the stock solution (1 in 10 [v/v] and 1 in 20 [v/v]). Results from specimens with ethanol alone were compared with the spiked samples. Recovery was found to be in the range 99 to 114%.

*Comparison with routine methods*

Results from the Advance immunoassay method were compared with those from a radioimmunoassay method (Amerlex cortisol kit, Amersham International PLC, Aylesbury, Buckinghamshire, UK). The Amerlex method was performed according to the manufacturer's instructions. In addition, results were compared with those from an HPLC technique.

In the HPLC technique the samples were first extracted using Clin. Elut CE1001 extraction columns (Analytichem International, Inc.). The following were applied, in order: 100 μl 0·1 mol/l hydrochloric acid; 100 μl 15 mg/l hexobarbitone in water; 500 μl cortisol standard (500 nmol/l in water) or sample; 400 μl water. Two 4 ml aliquots of dichloromethane were then allowed to percolate through each column and the effluents collected in conical glass tubes. The solvent was evaporated at 50 °C under a stream of air, and the residues re-dissolved in 100 μl of the mobile phase. 50 μl aliquots were then injected into the chromatograph.

The cortisol was quantitated by the method of peak height ratios using hexobarbitone as the internal standard.

*Conditions:*

| | |
|---|---|
| Mobile phase | 30% acetonitrile<br>70% 10 mmol/l potassium phosphate pH 3·2 |
| Column | 10 μm μBONDAPAK C-18 Radial-PAK (Waters Associates) and C-18 Guard-PAK |
| Flow rate | 3·0 ml/min |
| Detection | Absorbance at 242 nm; 0·005 AUFS. |

A total of 100 samples were analysed by each of the methods; not all were analysed by all three methods due to lack of sufficient sample. The data are shown in figures 1 and 2.

Regression parameters were obtained using the Deming procedure [6]. Results from calibrator cross-over studies are also included in the figures.

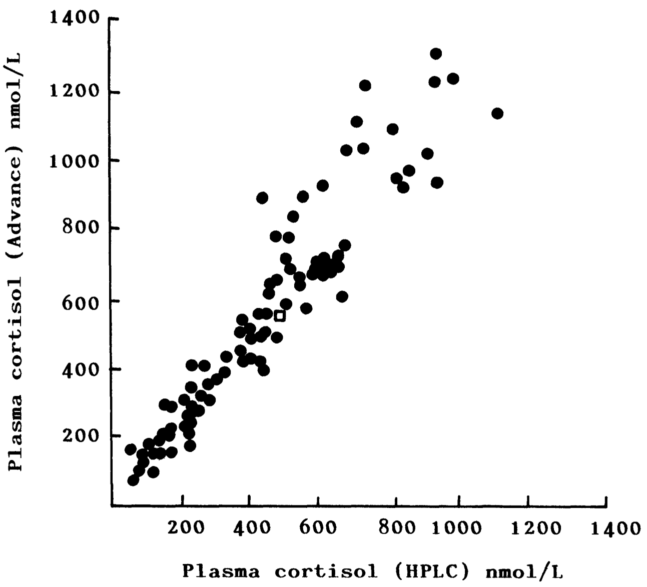

*Figure 1. Comparison of results for plasma cortisol by Advance and HPLC methods. Where $y = 1{\cdot}33x - 34{\cdot}58$, $r = 0{\cdot}944$, $N = 87$, SEM = 107 nmol/l. □ = HPLC calibrator.*

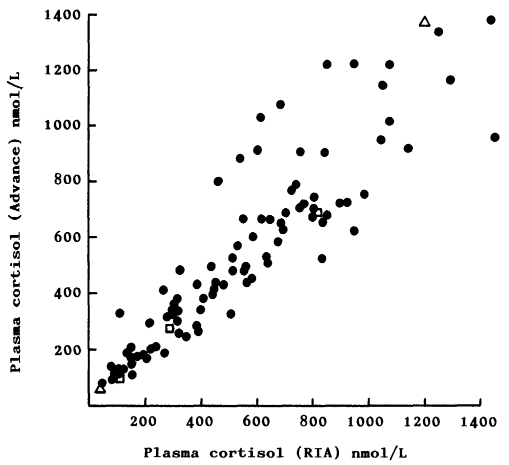

*Figure 2. Comparison of results for plasma cortisol by Advance and RIA methods. Where $y = 0{\cdot}97x + 10{\cdot}78$, $r = 0{\cdot}914$, $N = 95$, SEM = 137 nmol/l. △ = Advance calibrators and □ = RIA calibrators.*

*Quality assurance materials*

Examples of materials used in a cortisol external quality assessment scheme were assayed. The data compared with the Amerlex method consensus mean and also the GC-MS target value are given in figure 3.

One sample not included in the figure gave a grossly inaccurate result (Advance result = >1518 nmol/l, Amerlex consensus mean = 1155 nmol/l, GCMS target value = 274 nmol/l). This sample was known to contain prednisolone (2485 nmol/l).

*Calibration range and dilution curve*

A sample known to contain a level of cortisol in excess of the quoted range of the Advance system was diluted with

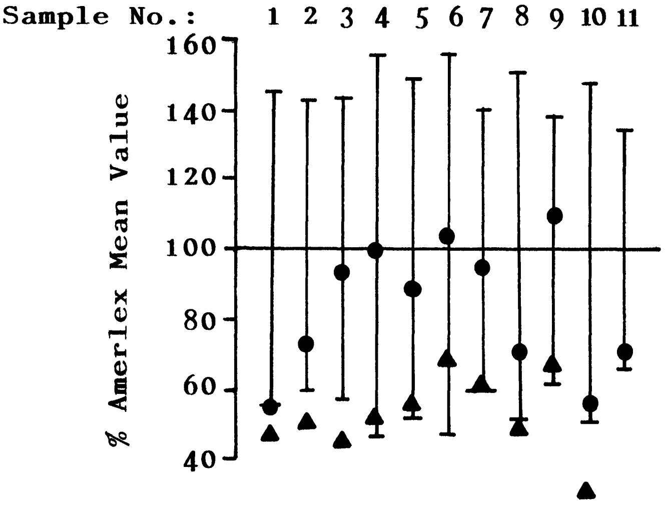

*Figure 3. Comparison of results obtained on samples from the external quality assurance scheme. Where ● = Advance result, ▲ = GC-MS target value, I = ±2 SDs of the consensus mean value for the Amerlex method. The mean values were (i) 359, (ii) 624, (iii) 928, (iv) 477, (v) 672, (vi) 685, (vii) 588, (viii) 394, (ix) 943, (x) 252, and (xi) 756 nmol/l.*

(1) 0·9% saline, and (2) a serum sample with a low cortisol level, to give a range of dilutions. The assay was shown to be linear over the range 400 nmol/l to 1400 nmol/l. Samples diluted to levels below 400 nmol/l gave results lower than the expected value.

*Detection limit*

An attempt was made to assess the potential detection limit of the assay by diluting the lowest calibrator 3 in 4, 1 in 2 and 1 in 4 (v/v). These dilutions were assayed together with ten replicates of a zero calibrator to determine the 'base-line noise'. The detection limit was taken to be the level of analyte giving a signal equivalent to the mean result for the zero calibrator minus three times the standard deviation determined within batch. The detection limit was 27 nmol/l.

*Stability of the calibration curve*

The stability of the calibration curve was assessed over a 24 h period. The system was calibrated at 9.00 h and low, medium and high controls analysed. This routine was repeated at 11.00, 13.00, 17.00, 21.00, 01.00, 05.00 and 09.00 h. The levels of the controls were then recalculated against the initial calibration and the results compared with the levels obtained using the adjacent calibration. The data are shown in figure 4.

*Kit stability*

(1) The kit stability over a three-month period was investigated. A calibration curve, 20 samples of a plasma pool (to assess precision) and a dilution curve were run using freshly reconstituted reagents. These reagents were stored at 4 °C and the protocol repeated at six and 13 weeks. Results obtained at week 13 were compared with those obtained with a new kit. The data are shown in table 2.

(2) The quenching rates for the low and high calibrators were recorded (in duplicate) and the differences calculated over a three-month period. The separation between high and low calibrators did not deviate outside the range of 93·4–113% of the mean value for the first week.

### Investigation of potential interferences

*Hyperbilirubinaemia*

The potential interferences due to hyperbilirubinaemia was investigated by spiking a plasma pool with solutions of bilirubin. A stock solution of bilirubin (Sigma Chemical Company, Poole, Dorset, UK) in dimethylsulphoxide (6000 µmol/l) was prepared. Doubling dilutions of the stock solution were made and added to pooled serum containing the analyte of interest (1 in 10 [v/v] dilution). A sample of the pooled plasma with DMSO alone was also assayed. The data are shown in figure 5.

*Table 2. Data to show the performance of a cortisol kit over a three-month period after reconstitution.*

| Parameter | | Fresh kit | Kit after six weeks | Kit after 12 weeks | New kit |
|---|---|---|---|---|---|
| Fluorescence rate | | 420·5 | 393·6 | 374·2 | 415·5 |
| Low calibrator | | 419·8 | 387·0 | 374·4 | 414·4 |
| Fluorescence rate | | 239·3 | 202·8 | 184·9 | 226·3 |
| High calibrator | | 239·4 | 201·3 | 188·8 | 226·9 |
| 'Separation' | | 181 | 188 | 187 | 188 |
| Precision $N = 20$ (CV%) | | 3·5% | 3·3% | 1·7% | 2·6% |
| Mean value µmol/l) | | 775 | 844 | 839 | 775 |
| Dilutions of high concentration specimen | Neat | >1518 | >1518 | >1518 | >1518 |
| | 5 + 1 | >1518 | >1518 | >1518 | 1512 |
| | 4 + 2 | 1292 | 1413 | 1250 | 1143 |
| | 3 + 3 | 880 | 1013 | 972 | 916 |
| | 2 + 4 | 549 | 629 | 668 | 569 |
| | 1 + 5 | 240 | 293 | 309 | 265 |

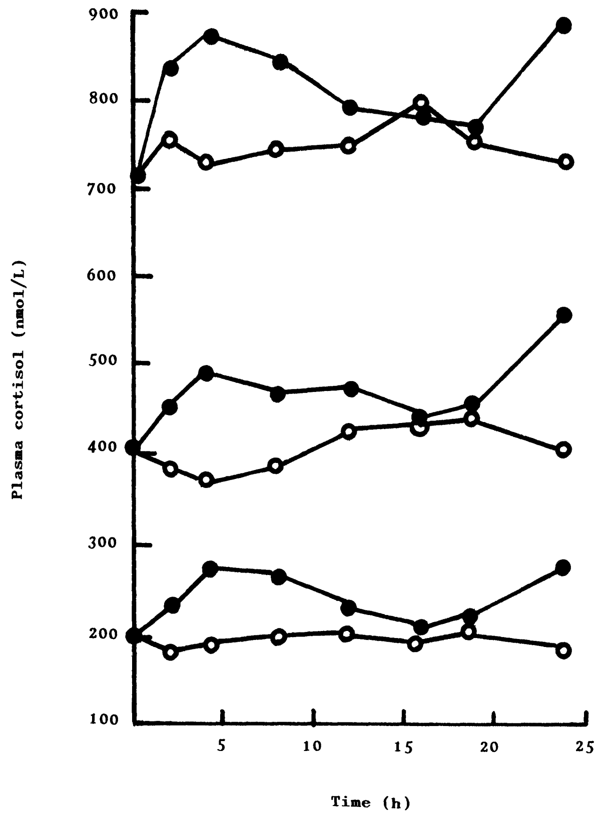

*Figure 4. Assessment of the frequency of calibration for the cortisol assay. Results calculated using the initial calibration (—●—) and the adjacent calibration (—○—).*

### Haemolysis

A sample (10 ml) of blood was collected from a volunteer, centrifuged and the red cells washed with isotonic saline. The red cells were then lysed by resuspension in deionized water and the solution adjusted with saline to give a haemoglobin concentration of 5·0 g/dl. This stock solution was diluted to give a range of haemoglobin concentrations. A plasma sample was spiked with the haemoglobin solutions (1 in 10 [v/v]) and cortisol measured. The data are shown in figure 5.

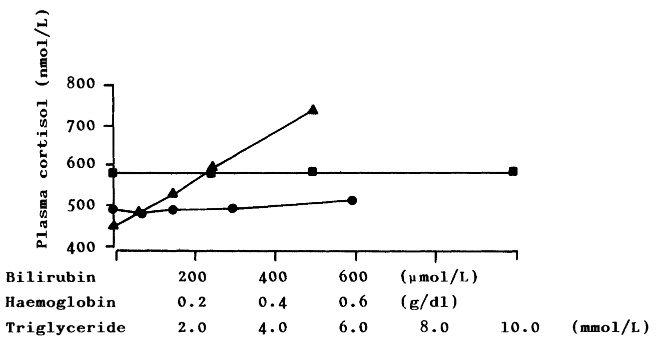

*Figure 5. Effect of bilirubin (—●—), haemoglobin (—▲—) and lipaemia (—■—) on the cortisol assay.*

### Lipaemia

The turbidity of pooled plasma was increased by addition of diluted Intralipid (Kabi Vitrum Ltd, Uxbridge, UK). The absorbance at 600 nm of a series of diluted specimens of known triglyceride level was used to assess the amount of Intralipid required. The data are shown in figure 5.

### Specific interferences

The following substances were investigated for potential interference in the cortisol assay; spironolactone, dihydroxyandrostenedione sulphate, cyproterone, 17-hydroxycyproterone, 17-hydroxyprogesterone, dexamethasone, 11-deoxycortisol and prednisolone. Each of the compounds was dissolved in ethanol to give stock solutions. Each potential interferent was then added (1 vol) to serum (9 vols); a control was prepared by substitution of the interferent by ethanol. The cortisol was then determined in each spiked sample and its control. The concentrations of metabolites were chosen to reflect the levels observed in pathological conditions. For the drugs, theoretical peak serum levels were calculated assuming complete absorption of the compound. Levels in excess of the theoretical peak level were added to the assay system. Prednisolone caused gross interference and was investigated at four concentrations. The data are shown in table 3.

*Table 3. Effect of several compounds on cortisol assay.*

| Compound | Concentration of interferent in sample | Cortisol measured (nmol/l) + compound | − compound |
|---|---|---|---|
| Spironolactone | 5·8 μmol/l | 610 | 617 |
| Dihydroxyandrostenedione sulphate | 10 μmol/l | 518 | 500 |
| Cyproterone | 1 μmol/l | 517 | 500 |
| 17 hydroxymetabolite of cyproterone | 1 μmol/l | 535 | 500 |
| 17 hydroxyprogesterone | 0·25 μmol/l | 519 | 500 |
| Dexamethasone | 510 nmol/l | 593 | 500 |
| 11-deoxycortisol | 570 nmol/l | 610 | 500 |
| Prednisolone | 1400 nmol/l | >1518 | 564 |
| | 700 nmol/l | >1518 | 564 |
| | 350 nmol/l | 1087 | 564 |
| | 175 nmol/l | 789 | 564 |

### Conclusion

The cortisol assay was simple to perform and the reagents were found to be stable for at least three months, after reconstitution. The precision of the assay compared favourably with the radioimmunoassay presently in use in the evaluator's laboratory. The '24 h calibration study' suggested that calibration on every carousel is advisable.

The accuracy of the method judged by recovery of analyte and analyses of quality assurance materials was satisfactory. In the case of the EQA samples, the results were generally lower than the consensus mean values for the Amerlex users, lying between this value and the GCMS target value. Comparison of results with those obtained using RIA yielded a wide scatter, although the regression parameters gave a slope of 0·97; it should be noted that no attempt was made to screen samples for the presence of interferents, such as prednisolone. It is assumed that the discrepant results are due to differing specificities for the antibodies used. Comparison with HPLC indicated a significant bias; reflecting the bias seen with the Advance results in EQA materials when compared with the GCMS target values.

Hyperbilirubinaemia (up to 600 μmol/l) and lipaemia (up to 10 mmol/l triglyceride) did not affect the assay. Haemolysed specimens give inaccurate results (falsely high) due to quenching of the fluorescence rates by haemoglobin. Severe interference was noted in the case of prednisolone. It is debatable whether this is important, in that it might be claimed that the clinician will always be aware if a patient is taking prednisolone—however, this information may not always reach the laboratory and the appropriate warning may not be given.

### Acknowledgements

We would like to thank Dr G. Groome for supplying materials from the Cardiff external quality assessment scheme.

### References

1. Förster, T., *Ann. Physik.*, **2** (1948), 55.
2. Ullman, E. F., Bellet, N. F., Brinkley, J. M. and Zuk, R. F., Laboratory and research methods in biology and medicine, Vol. 4. *In Immunoassays: Clinical Laboratory Techniques for the 1980's*, R. M. Nakamura, W. R. Ditto and E. S. Tucker III (Eds) (A. R. Liss, Inc., New York, 1944, 13–43.
3. Guilbault, G. G. (Ed), *Practical Fluorescence. Theory, Methods and Techniques* (Marcel Dekker, Inc., New York, 1973), 521–541.
4. Ullman, E. F., and Khana P. L., *Methods in Enzymology* **74(C)** (1981), 28.
5. Dixon, K. *Annals of Clinical Biochemistry*, **19** (1982), 224.
6. Cornbleet, P. and Gochman, N., *Clinical Chemistry*, **25** (1979), 432.

### EASTERN ANALYTICAL SYMPOSIUM TO CELEBRATE SILVER JUBILEE

As reflected by a substantial rise in attendance over the last few years, the Eastern Analytical Symposium has established itself as a significant national and international event in the scientific community.

1986 will mark the 25th annual EAS, which has been christened the Silver Jubilee. To mark this occasion, the EAS Governing Board has authorized the first-ever five-day EAS. A total of 50 technical sessions are planned for the five days of the meeting, as well as the traditional EAS poster sessions which will be held each day, giving a substantial increase in the number of papers to be presented at the Silver Jubilee.

In conjunction with the celebration of the Silver Jubilee, the EAS will present the First Eastern Analytical Symposium Award for Outstanding Contributions to the Fields of Analytical Chemistry.

The Silver Jubilee will be the first EAS to take place at the New York Hilton Hotel; the facilities at the Hilton will permit the expansion of the exposition to accommodate a greater variety of exhibits than has heretofore been possible, while permitting the exposition to take place in a location exceptionally convenient to all meeting rooms.

With the move to the Hilton, the EAS has been able to finalize its meeting dates for the next five years—

1986: October 6–October 10
1987: September 14–September 18
1988: September 26–September 30
1989: September 24–September 28
1990: September 24–September 28

*Details (exhibiting and attending) from Dr S. David Klein, EAS Publicity, 642 Cranbury Cross Road, North Brunswick, New Jersey 08902, USA. Tel.: 201 846 1582.*

